# Waste-Based Pigments for Application in Ceramic Glazes and Stoneware Bodies

**DOI:** 10.3390/ma12203396

**Published:** 2019-10-17

**Authors:** Jorge Carneiro, David Maria Tobaldi, Marinélia Neto Capela, Maria Paula Seabra, João António Labrincha

**Affiliations:** Department of Materials and Ceramic Engineering, CICECO, University of Aveiro, 3810-193 Aveiro, Portugal; david.tobaldi@ua.pt (D.M.T.); marinelia.capela@ua.pt (M.N.C.); jal@ua.pt (J.A.L.)

**Keywords:** granite sludge, electroplating sludge, marble sludge, pigments, waste valorization

## Abstract

The use of wastes, some of them hazards, as raw materials of ceramic pigments has been a way to diminish their environmental impact, to economically valorize them, and to face the depletion of virgin raw materials. In this work were prepared pigments having in their composition only industrial wastes: Cr/Ni electroplating (ES), and sludges from the cutting of natural stones—marble (MS) and granite (GS). The prepared mixtures were calcined at three temperatures (1100, 1200, and 1300 °C) and the obtained powders were characterized by XRD and UV-vis. Their coloring strength and thermal stability were assessed by adding them to different ceramic substrates: glazes (transparent bright and opaque matte) and a stoneware paste. The CIEL*a*b* coordinates of the fired materials were measured. The developed pigments are thermally stable and exhibit good tinting power, originating nicely colored and defect-free ceramic materials.

## 1. Introduction

The consuming model of the modern society produces more and more wastes that are no longer useful. In particular, the industrial sector generates huge amounts of waste, reaching levels that are not sustainable. Thus, the growing level of environmental awareness has put waste management as one of the key challenges of our society. The concept of waste valorization, i.e., transforming wastes into raw materials or products, has, therefore, gained relevance. This idea fits with the circular economy concept that is an economic system aimed at eliminating waste and the continual use of virgin raw materials. Circular systems employ recycling, reuse, remanufacturing, and refurbishment to create a closed system, minimizing the use of resource input and the creation of waste [1].

Inorganic pigments—high added-value products—are materials currently used in several sectors, namely in the ceramic and glass industry. These materials must fulfil three fundamental requirements: to have a high coloring power, to be thermally stable at high temperatures, and to be chemically stable when fired with the glazes or the ceramic bodies [2]. 

The use of industrial wastes, some of them hazardous, to synthetize inorganic pigments for ceramic applications has been a topic explored in the last years [3,4,5,6,7,8,9,10,11,12,13]. Some examples of industrial wastes used as secondary raw materials for the pigment’s synthesis include: titanium slag [3], ashes from anaerobic treatment of municipal sewage [4], electroplating sludge [5,6,7,8,9], leather sludge [10,11], red mud [7,12], and rice husk biomass [13].

In this work, wastes from different industrial sectors: a sludge from electroplating process (ES—rich in Cr and Ni), a sludge from the marble sawing (MS—rich in CaCO_3_) and a sludge from the granite sawing (GS—rich in SiO_2_), were used to produce inorganic pigments. Their combination, in the right proportions, will generate stable pigment structures where the hazardous elements will be immobilized. The economic value cannot be understated as well, since all the wastes used in this work represents an extra cost to the respective producers. The approach followed in this work is focused on the synthesis of ceramic pigments using only wastes, with no addition of virgin raw materials to adjust/enhance the pigment properties. The production route (aiming for a future scale-up) is simple to implement considering the existing industrial processes.

## 2. Experimental Details

### 2.1. Materials

Electroplating sludge (ES) was provided by a Cr/Ni planting industry (Ghroe, Portugal) and is classed as hazardous waste. The marble sludge (MS) was collected from CEVALOR (Technological Centre for the Utilization and Valorisation of Ornamental and Industrial Stones), while the granite sludge (GS) was produced by Euro Granitos. Both are classed as inert wastes, but their production rates are very significant. Esmalglass-Itaca group provided the glazes (bright lead-free transparent and opaque matte) and GRESTEL S.A. provided the stoneware paste.

### 2.2. Pigments Preparation

ES, MS and GS were mixed in different weight proportions: GS:ES (50:50, 25:75 and 75:25) and GS:MS:ES (30:20:50)—see Table 1. The homogenization was done by wet ball milling. Afterwards, the mixtures were dried in a ventilated oven at 80 °C. The obtained powders were fired, in an electric furnace under a static airflow, with a heating rate of 5 °C/min until the maximum temperature (1100 °C, 1200 °C and 1300 °C) and a dwell time of 3 h. Until 550 °C, the cooling rate was also 5 °C/min. The obtained pigments were then ball milled with water for 1 h at 300 rpm, dried (80 °C) and sieved below 63 µm.

### 2.3. Coloring Potential and Stability

An amount of 3 wt.% of each pigment was added to three types of ceramic supports: a transparent and bright lead-free glaze (TB), an opaque matt glaze (OM) and a stoneware ceramic body (SB). The influence of the pigment content was tested in the OM support to which two other distinct amounts were added (5 and 10 wt.%). The homogenization process of the pigment with the ceramic substrate was conducted by wet ball mixing. The mixtures were dried at 80 °C, disaggregated, and sieved (at 63 µm). The obtained powders were pressed into Ø 2.5 cm pellets and fired in an electric furnace in air. For the glazes (TB and OM) the heating and cooling rates were 10 °C/min and the soaking time at the maximum temperature (1100 °C) was 30 min. For the mixtures of pigments with SB the maximum firing temperature was 1200 °C and the soaking time was also 30 min. The heating rate was 5 °C/min but two dwells of 30 min were performed at 575 °C and 770 °C. The cooling rate was 10 °C/min. To assess the color stability of the prepared pigments with the temperature the mixtures with TB were also fired at 1085 °C and 1115 °C (1100 ± 15 °C).

### 2.4. Sample Characterization

The chemical composition of the wastes (RM, ES and GS) was obtained by X-ray fluorescence (X’Pert PRO MPD spectrometer, Malvern PANalytical, Almelo, The Netherlands). The loss on ignition (LOI) at 1000 °C was also determined. The particle size distribution was determined by laser diffraction on a Coulter LS particle size analyzer (LS230FM, Beckman Coulter, Brea, CA, USA). Their mineralogical composition was assessed by X-ray diffraction (XRD). XRD was also used to qualitatively determine the crystalline phases present in the prepared specimens. XRD for semi-quantitative phase analysis (QPA) was performed on selected specimens. All of the XRD patterns were recorded at room temperature on a θ/θ diffractometer (X’Pert Pro, Malvern PANalytical, Almelo, The Netherlands), equipped with a fast RTMS detector (PIXcel 1D, Malvern PANalytical, Almelo, The Netherlands), with Cu *K*_α_ radiation (45 kV and 40 mA, 15°–80° 2θ range, with a virtual step scan of 0.02° 2θ, and virtual time per step of 200 s). QPA was assessed on selected specimens, by way of the Rietveld method [14] on the XRD data; Rietveld analysis was performed *via* the GSAS-EXPGUI software package [15].

The optical properties were measured by absorption spectroscopy (UV-3100 UV–Vis–NIR spectrometer, Shimadzu, Kyoto, Japan, in the UV-vis range 300–850 nm), whilst the L*a*b* color coordinates were extracted by means of a portable colorimeter—Chroma Meter CR-400 (Konica Minolta, Tokyo, Japan)—using D_C_ illuminant and 10° standard observer (Y: 94.0, x: 0.3130, y: 0.3191) according to the Commission Internationale de l’Eclairage (CIE). CIE L*a*b* data are expressed as brightness L*, changing from 0 (black) to 100 (white), a*(+red, −green), and b*(+yellow, −blue) [16]. The color stability was calculated using the value of ∆E: $\Delta E=\sqrt{{\left(\Delta L\right)}^{2}+{\left(\Delta a\right)}^{2}+{\left(\Delta b\right)}^{2}}$. Values of ∆E > 2 mean that the color change can be noticed by the naked eye [17].

## 3. Results and Discussion

### 3.1. Wastes Characterization

The chemical composition of the wastes is presented in Table 2. ES is mainly composed by: nickel (25.86 wt.%), chromium (15.29 wt.%), sulphur trioxide (5.84 wt.%), silica (5.63 wt.%), and phosphorus pentoxide (4.45 wt.%). This sludge exhibits a high (34.30 wt.%) LOI value. On the contrary, GS presents a small LOI value (1.50 wt.%) being silica (64.12 wt.%) and alumina (17.94 wt.%) the two main constituents. Smaller amounts of potassium (6.10 wt.%), sodium (3.10 wt.%), and iron oxide (3.38 wt.%) are also present. Lastly, MS is calcareous-rich: calcium oxide (56.56 wt.%), while LOI = 41.95 wt.%. A pure calcium carbonate shows about 44% of LOI, meaning that the MS contains about 95% of such component.

The particle size distribution of the starting materials is a characteristic that strongly affect their reactivity. Moreover, the granulometric distribution can also influence the mixture homogeneity. The ES sludge outcomes from the flocculation process that occurs during the wastewater treatment in the Cr/Ni electroplating plant. As reported in previous works of the authors [7] the non-calcined ES has an average particle size of ≈112 µm and a wide size range (from sub-micron sizes to ≈500 µm) due to the presence of large agglomerates. The other two sludges exhibit a much narrow distribution and a smaller medium particle size (22.3 µm and 4.6 µm, respectively, for GS and MS).

**Figure 1:**
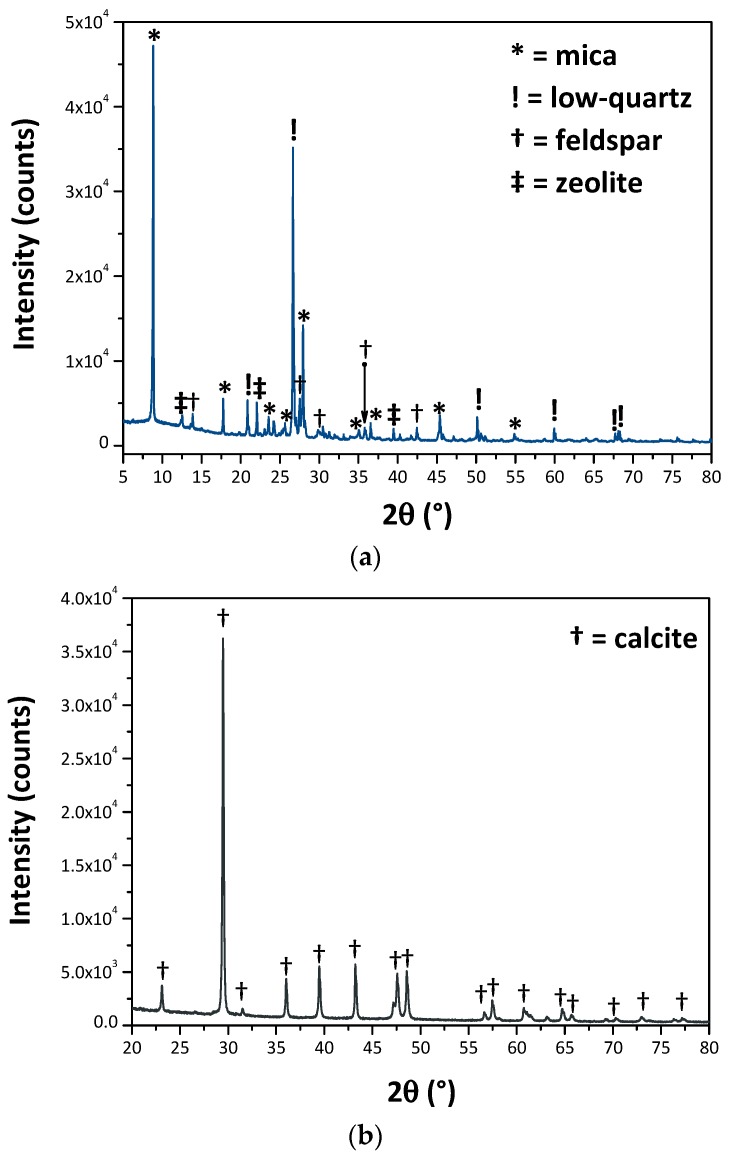

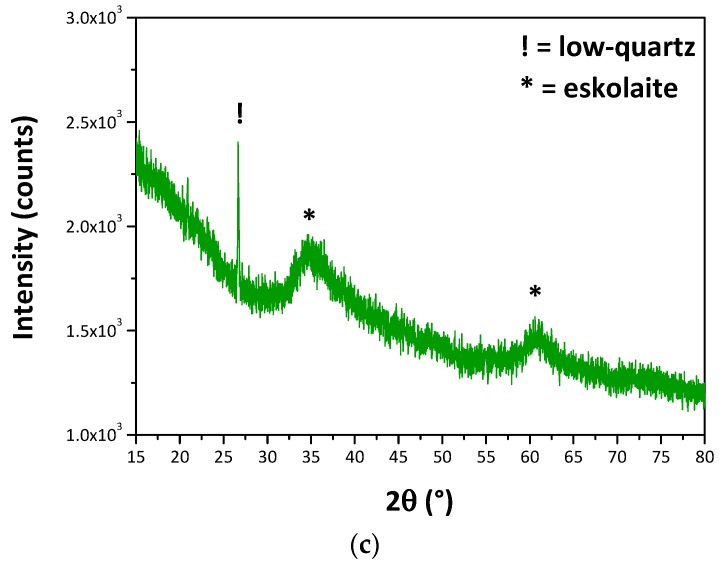
XRD patterns of: (**a**) GS; (**b**) MS and (**c**) ES.

The mineralogical composition of the wastes has been revealed by XRD, Figure 1a–c. GS (Figure 1a) is mainly (qualitatively) composed of low-quartz, a mica group mineral, a feldspar, and a zeolite group mineral as well—this latter likely coming from atmospheric weathering of the granite. As suggested before, MS is composed by calcium carbonate, as all of the reflections are assignable to CaCO_3_ (Figure 1b), whilst GS showed itself to have a very low crystalline nature, the only crystalline phases detected being low-quartz, and eskolaite (Cr_2_O_3_), as shown in Figure 1c [7].

### 3.2. Pigments Characterization

**Figure 2:**
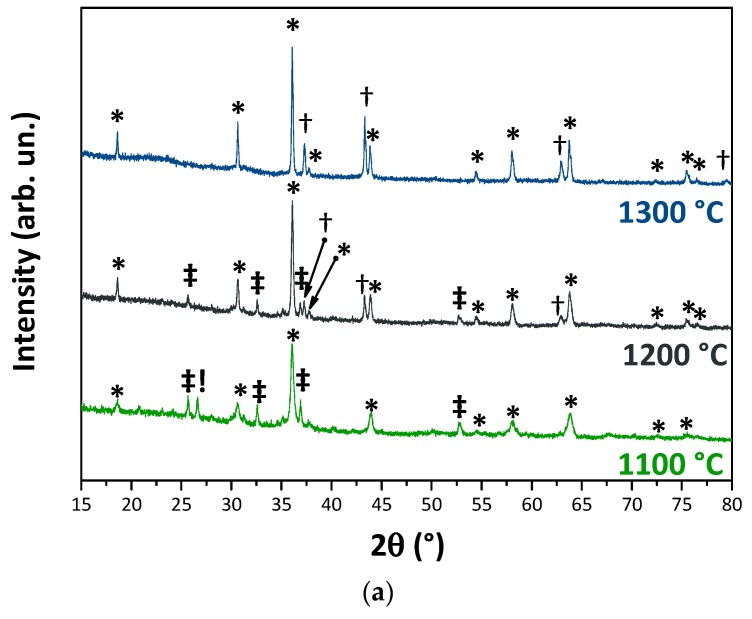

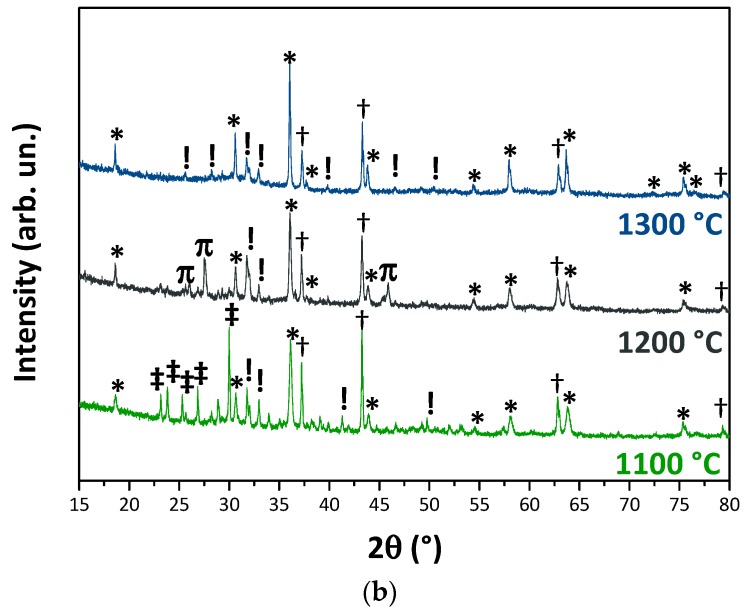
XRD patterns of 50GS:ES (**a**) and 30MS:20GS:ES (**b**) after the thermal treatment at 1100 °C, 1200 °C and 1300 °C. Symbols in (a) * = spinel; † = NiO; ‡ = Ni_2_SiO_4_; ! = low-quartz. Symbols, in (b) * = spinel; † = NiO; ‡ = wollastonite; π = pseudowollastonite; ! = alite.

The temperatures at which the prepared mixtures were fired (1100, 1200 and 1300 °C) were selected considering results of previous works [7,12]. XRD patterns of the formulations 50GS:ES and 30GS:20MS:ES after thermal treatment at such temperatures are shown in Figure 2a,b. When GS and ES are mixed in equal wt.% amounts, and thermally treated at high temperatures, both the color and the mineralogy changes, Figure 2a. At 1100 °C, the main crystalline phases detected are: (i) a mineral of the spinel group; (ii) nickel oxide (bunsenite); (iii) a mineral of the olivine group (tentatively: Ni_2_SiO_4_); (iv), and low-quartz. Increasing the thermal treatment temperature to 1200 °C, the residual low-quartz disappeared, probably enriching the glassy phase. At this isotherm, the mineralogical phases are: (i) the spinel; (ii) the mineral of the olivine group; and (iii) NiO. At the highest temperature, 1300 °C, spinel (68.7 wt.%) and NiO (31.3 wt.%) are the only two crystalline phases, as listed in Table 3.

**Figure 3:**
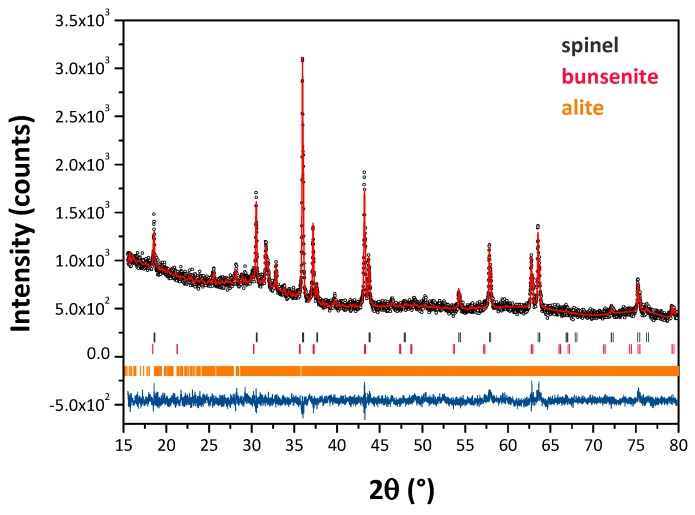
Graphic output of the Rietveld refinement of specimen 30MS:20GS:ES_1300. The red continuous line represents the calculated pattern, the black open circles represent the observed pattern, and the difference curve between observed and calculated profiles is plotted below. The position of reflections is indicated by the small vertical bars (black: spinel; red: bunsenite; orange: alite).

Additions of MS into the system lead to differences on the color and mineralogy (Figure 2b and Figure 3). At the lowest firing temperature, specimen 30MS:20GS:ES is composed of spinel, NiO, wollastonite and alite. Those two latter mineralogical phases result from the addition of calcium to the system. At the intermediate firing temperature of 1200 °C, we assist at the disappearance of wollastonite at the expenses of an increase in the alite content, together with the crystallization of pseudowollastonite. At 1300 °C, the pigment is (semi-quantitatively) composed of: 35.3 wt.% spinel, 18.0 wt.% NiO, and 46.7 wt.% alite—cf. Table 3, and as graphically reported in Figure 3.

**Figure 4:**
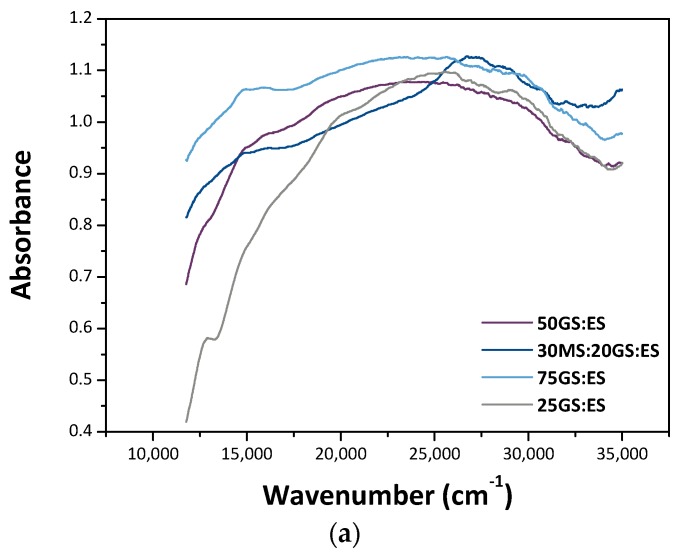

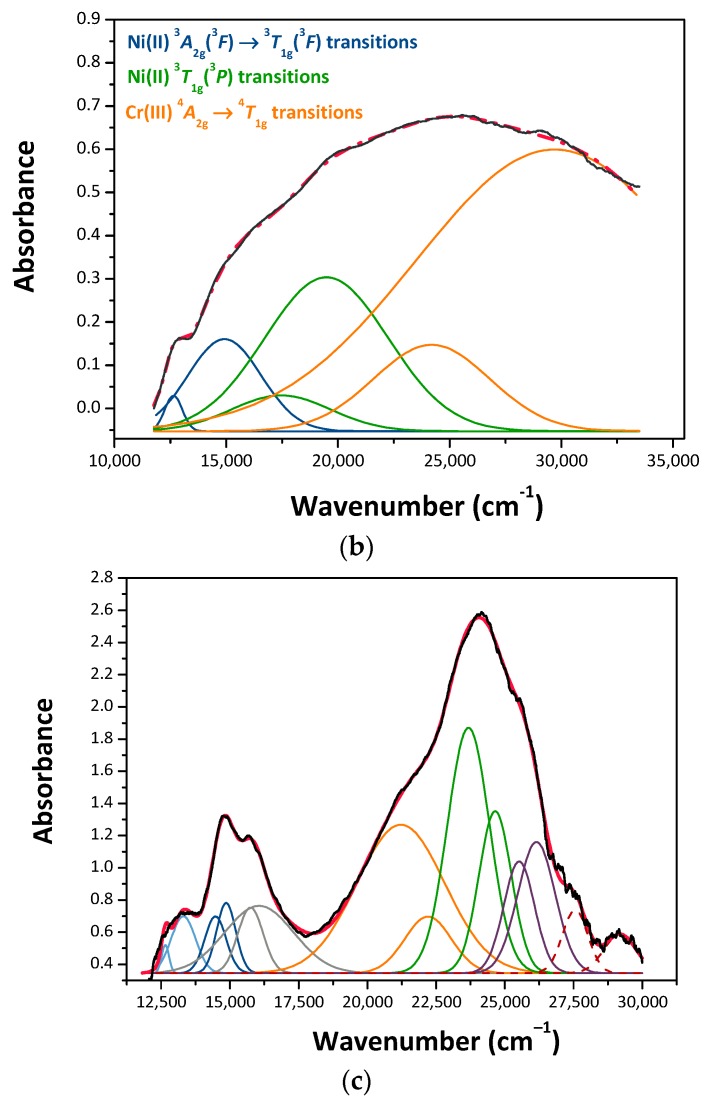
(**a**) Optical spectra of specimens thermally treated at 1300 °C (wavenumber Vs absorbance). (**b**) Optical spectrum of specimen 25GS:ES_1300 in which the deconvoluted optical bands are reported. In it, the black continuous line represents the recorded data, the dash-dotted red line is the cumulative peak fit. (**c**) Optical spectrum of specimen ES fired at 1200 °C (modified from Reference [7]).

Optical spectra of all the specimens fired at 1300 °C are depicted in Figure 4a. They all show similar optical characteristics, with the exception of specimen 30MS:20GS:ES_1300, in which a drop-in absorption in the 25,000–15,000 cm^−1^ region, due to the non-negligible presence of Ca_3_SiO_5_, is detectable, cf. Table 3. In this work, we will describe in detail only the optical spectrum of 25GS:ES_1300, Figure 4b. The UV-vis spectrum of this specimen is dominated by Cr^3+^ and Ni^2+^ optical signatures. The bands at low energy, in blue in Figure 4b, can be tentatively assigned to Ni(II) ^3^*A*_2g_(^3^*F*)→^3^*T*_1g_(^3^*F*) transitions [18]. In addition, the weak bands at ~17,500–20,000 cm^−1^ (bands in green in Figure 4a) belong to Ni(II) ^3^*T*_1g_(^3^P) transitions [18]. The bands at higher energy (those in orange in Figure 4b) are tentatively assigned to Cr(III) ^4^*A*_2g_→^4^*T*_1g_ electronic transitions [19]. The signal at high energy (~35,000 cm^−1^) is due to charge transfers between metals and the oxygen ions [20]. The UV-vis spectrum of specimen ES fired at 1200 °C is also reported for comparison (Figure 4c). In it, the bands in light blue were tentatively assigned to Ni(II) ^3^*T*_1g_(^3^*F*) transitions; those in dark blue to ^3^*A*_2g_(*F*)→^3^*T*_1g_(*F*) transitions of Ni(II); those in orange to Ni(II) ^3^*T*_1g_(^3^*P*) transitions, and those in violet to Ni(II) ^1^*T*_2g_ transitions [7]. Absorbance bands in light grey and green are tentatively assigned to Cr(III) transitions: ^4^*A*_2g_→^4^*T*_2g_ and ^4^*A*_2g_→^4^*T*_1g_(^4^*F*), respectively [7].

### 3.3. Color Development in Ceramic Bodies

As already said, the coloring hue and tinting power of the prepared waste based-inorganic pigments was evaluated by adding them to a transparent and bright lead-free glaze [TB: SiO_2_ (51–53 wt.%); Al_2_O_3_ (41–43 wt.%); ZrO_2_ (7–9 wt.%); P_2_O_5_ (1–3 wt.%)], an opaque matt glaze [OM: SiO_2_ (54–56 wt.%); Al_2_O_3_ (20–22 wt.%); CaO (10–12 wt.%); K_2_O (3–5 wt.%); Na_2_O (3–5 wt.%); MgO (2–4 wt.%)], and a stoneware ceramic body (SB).

**Figure 5:**
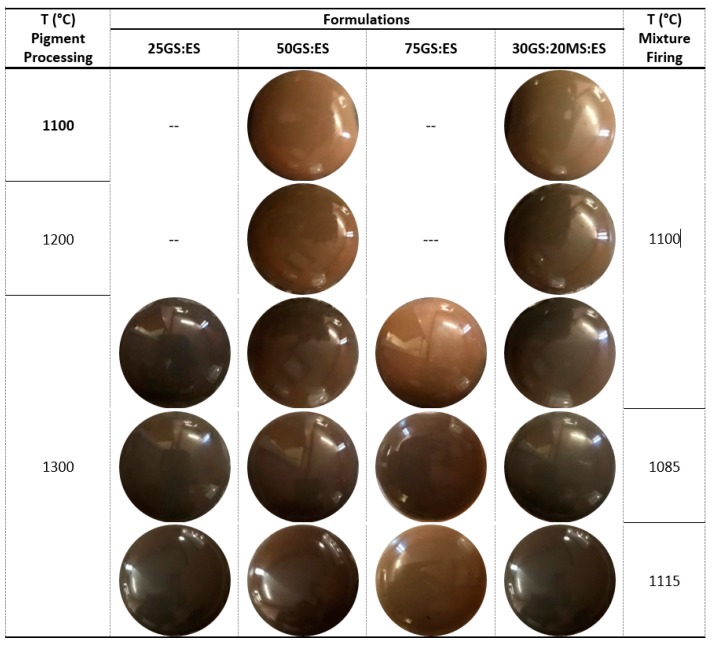
Transparent and bright lead-free glaze loaded with 3 wt.% of waste-based ceramic pigments (25GS:ES, 50GS:ES, 75GS:ES and 30GS:20MS:ES processed at different temperatures) fired at 1100 ± 15 °C.

Figure 5 presents the images of the TB glaze colored samples; the measured CIEL*a*b* color coordinates are patent in Table 4. The processing temperature of the pigments has a notorious influence in the developed color for both tested formulations: 50GS:ES and 30GS:20MS:ES. This was expected from changes on mineralogy (Figure 2). In general, there is a darken tendency when processing temperature rises, as normally happens in all ceramic systems. With 50GS:ES formulation, the temperature rises from 1100 to 1300 °C promotes a decrease of the L* value from 47.2 to 36.8 (≈22%), a* from 3.7 to 2.3 (≈38%) and b* from 14.3 to 5.5 (≈61%). The pigment containing the three studied wastes—30GS:20MS:ES—exhibits a similar behavior; however, the influence of the firing temperature is more pronounced. The decrease of L* is ≈18% (from 45.7 to 37.3), a* ≈ 58% (from 1.2 to 0.5) and b* ≈ 71% (from 11.1 to 3.2).

At 1300 °C, the growth of ES percentage (from 25 wt.% to 75 wt.%)—formulations 75GS:ES, 50GS:ES and 25GS:ES—intensified the hue of the pigment. TB mixtures exhibits a decrease of L* (from 45 to 36.8), a* (3.9 to 1.2) and b* (12.7 to 3.4) values, which is in accordance with the increase of the spinel phase amount (see Table 3).

The partial substitution of GS by MS, keeping constant the ES amount—compositions 50GS:ES and 30GS:20MS:ES—and the processing temperature (1300 °C), did not promote a significant alteration of the pigments coloring power (similar L* value). However, these two pigments do not have the same crystalline composition (see Table 3), what is reflected in the developed color—a* and b* values exhibit a variation of 58% and 85%, respectively.

The color stability was accessed on formulations processed at 1300 °C by varying 30 °C (1100 ± 15 °C) the firing temperature of the mixture TB loaded with 3 wt.% of pigment. The obtained results (Table 4) show that all of them have a very good thermal stability; ΔE values are always lower than 2, meaning that the color change cannot be noticed by the naked eye [17]. This is a consequence of the formation of relatively stable crystalline phases within that temperature range (see Table 3).

**Figure 6:**
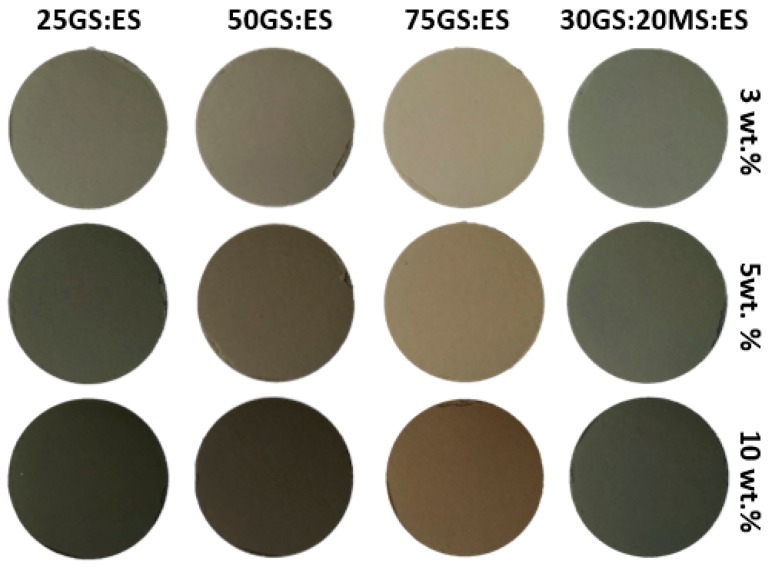
Opaque matte (OM) glaze loaded with different amounts (3, 5, and 10 wt.%) of ceramic waste-based pigments fired at 1300 °C.

The OM glaze incorporated three distinct amounts (3, 5, and 10 wt.%) of all the pigments processed at 1300 °C. The aspect of the obtained samples and their CIEL*a*b*coordinates are patent in Figure 6 and Table 5, respectively. The increase of the pigment amount enhances the darkness of the samples, but the hue remains almost unchanged: a* and b* color coordinates do not exhibit significant changes; only the L* value exhibits a notorious decrease (between 27% and 29%).

**Figure 7:**
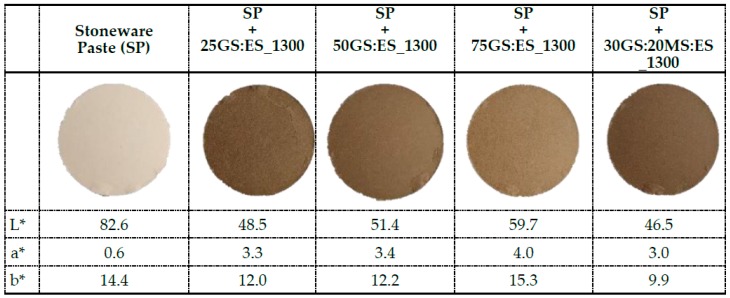
Stoneware paste (SP) without and loaded with 3 wt.% of the prepared waste-based pigments processed at 1300 °C.

Since are appearing new markets for products made with colored stoneware pastes, namely household and decorative stoneware pieces, their production has been gradually growing. Consequently, the consumption of ceramic pigments for this type of application has been increasing as well and the actual waste-based pigments were also tested for this purpose. The aspect and the CIEL*a*b* color coordinates of the stoneware paste without and with 3 wt.% of pigments (fired at 1300 °C) is presented in Figure 7.

The addition of the developed pigments to the stoneware paste did not induce the appearance of defects neither change of the material’s technical properties allowing to obtain nice colored stoneware bodies. The pigments made from GS- and ES-originated colors from dark beige (camel) to light brown, tendentiously darker with increasing amounts of ES. The 30GS:20MS:ES_1300 pigment also originates light brown hues (Figure 7), but darker than those obtained with the binary mixtures. This behavior is opposite to that observed on glazes (TB and OM). Finally, we should mention that the addition of the actual pigments did not induce the appearance of defects neither changes on the technical properties allowing to obtain nice colored stoneware bodies.

## 4. Conclusions

This work proved that the production of stable inorganic pigments only from wastes is feasible. Binary and ternary combinations were validated: (i) granite sludge (GS) and electroplating sludge (ES); and (ii) GS + ES + marble sludge (MS).

Three processing temperatures (1100, 1200 and 1300 °C) were tested and it was concluded that firing at highest temperature is advisable to ensure higher chemical and thermal stability. After being fired at 1300 °C the crystalline phases present in the mixtures of GS and ES (25GS:ES, 50GS:ES and 75GS:ES) are a spinel and NiO. A small amount of *α*-Fe_2_O_3_ (2.9 wt.%) was detected in the composition with the higher amount of GS (75GS:ES). The partial substitution of GS by MS (30GS:20MS:ES) leads to the formation of another crystalline phase—Ca_3_SiO_5_. In general, darker hues are obtained when the ES content increases.

The UV-vis spectrum of the prepared pigments presents three bands at approximately 12,500 cm^−1^, 20,000 cm^−1^ and 25,000 cm^−1^ which are assigned, respectively, to Ni^2+ 3^*T*_1g_(^3^F), Ni^2+ 3^*T*_1g_(^3^P) and Cr^3+ 4^*A*_2g_→^4^*T*_1g_(^4^F) electronic transitions.

The developed waste-based pigments are suitable for coloring glazes (transparent bright lead free and opaque matte) and stoneware pastes. The developed hues change with the substrate nature. In bright and transparent lead-free glaze, the pigments developed colors that change from light camel to dark grey. In the case of the opaque matt glaze, hues fluctuate from beige to dark grey. In a stoneware paste, the achieved colors oscillate between camel and light brown. All the prepared colored products are free of defects and show suitable technical properties. This confirms the chemical and thermal stability of the prepared pigments. In transparent lead-free glaze, color changes induced by temperature variations (1100 ± 15 °C) are not detected by the naked eye (ΔE < 2).

## Figures and Tables

**Table 1:** Prepared compositions and maximum processing temperatures.

Sample ID	Compositions (wt.%)	Max. Processing Temperature (°C)
	GS:ES	
25GS:ES_1300	25:75	1300
50GS:ES_1100	50:50	1100
50GS:ES_1200		1200
50GS:ES_1300		1300
75GS:ES_1300	75:25	1300
	MS:GS:ES	
30MS:20GS:ES_1100	30:20:50	1100
30MS:20GS:ES_1200		1200
30MS:20GS:ES_1300		1300

**Table 2:** Chemical composition of the dried granite sludge (GS), marble sludge (MS) and electroplating sludge (ES) obtained by X-ray fluorescence (XRF).

Component (wt.%)	GS	MS	ES
Na_2_O	3.10	0.03	-
MgO	0.65	0.34	0.16
Al_2_O_3_	17.94	0.19	1.66
SiO_2_	64.12	0.34	5.63
P_2_O_5_	0.76	0.02	4.45
SO_3_	0.36	0.21	5.84
K_2_O	6.10	0.02	0.07
CaO	1.12	56.56	1.09
Fe_2_O_3_	3.38	0.07	0.65
Cr	–	–	15.29
Ni	–	–	25.86
Cu	–	–	2.23
Zn	0.01	–	1.58
LOl	1.50	41.95	34.30

**Table 3:** Quantitative phase composition as derived from the Rietveld refinements.

Sample ID	Phase Composition (wt.%)*			
	Spinel	NiO	Ca_3_SiO_5_	*α*-Fe_2_O_3_
50GS:ES_1300	68.7 ± 0.1	31.3 ± 0.2	–	–
30MS:20GS:ES_1300	35.3 ± 0.2	18.0 ± 0.2	46.7±0.5	–
75GS:ES_1300	62.7 ± 0.1	34.4 ± 0.2	–	2.9±0.2
25GS:ES_1300	93.7 ± 0.1	6.3 ± 0.5	–	–

* There were 4910 total observations for each refinement. The agreement factors of all the refinements were: *χ*^2^ ≤ 1.77; *R*_F_^2^ ≤ 11.81%; *R*_wp_ ≤ 4.86%; *R*_p_ ≤ 3.78%.

**Table 4:** CIEL*a*b* color coordinates of the transparent and bright (TB) lead-free glaze loaded with 3 wt.% of waste-based pigments (the mixtures were fired at 1100 ± 15 °C).

Pigment ID	Firing Temperature(°C)	L* 0 (Black) to 100 (White)	a* (+Red, −Green)	b* (+Yellow, −Blue)	∆E
25GS:ES_1300	1085	44.2	4.0	12.0	1.1
	1100	45.0	3.9	12.7	--
	1115	45.3	4.0	13.6	1.0
50GS:ES_1100	1100	47.2	3.7	14.3	--
50GS:ES_1200		41.0	3.5	11.0	--
50GS:ES_1300	1085	36.4	2.1	4.5	1.0
	1100	36.8	2.3	5.5	--
	1115	35.8	2.4	3.4	0.8
75GS:ES_1300	1085	36.7	1.0	4.3	1.4
	1100	35.6	1.2	3.4	--
	1115	35.2	1.5	1.8	1.7
30MS:20GS:ES_1100	1100	45.7	1.3	11.1	--
30MS:20GS:ES_1200		39.0	0.9	6.6	--
30MS:20GS:ES_1300	1085	38.2	0.4	3.3	0.8
	1100	37.3	0.5	3.2	--
	1115	36.6	0.7	2.6	1.0

**Table 5:** CIE L*a*b* color coordinates of the opaque matt (OM) glaze loaded with different pigments formulations and amounts of waste-based pigments (fired at 1100 °C).

Pigment ID	Pigment Content (wt.%)	L* 0 (Black) to 100 (White)	a* (+Red, −Green)	b* (+Yellow, −Blue)
25GS:ES_1300	3	68.4	0.5	10.3
	5	60.9	1.4	10.8
	10	49.9	2.5	10.6
50GS:ES_1300	3	58.5	0.4	7.5
	5	51.3	0.6	6.9
	10	41.9	0.8	5.9
75GS:ES_1300	3	55.4	−1.4	6.6
	5	47.9	−1.2	5.8
	10	39.2	−0.9	4.5
30MS:20GS:ES_1300	3	59.3	−1.9	5.4
	5	51.3	−1.8	5.1
	10	41.9	−1.6	4.1
